# Catalytic properties of wheat phytase that favorably degrades long-chain inorganic polyphosphate

**DOI:** 10.5713/ajas.19.0047

**Published:** 2019-05-27

**Authors:** Jeongmin An, Jaiesoon Cho

**Affiliations:** 1 Department of Animal Science and Technology, Konkuk University, Seoul 05029, Korea

**Keywords:** Animal Husbandry, Exopolyphosphatase, Inflammation, Inorganic Polyphosphate, Public Health, Wheat Phytase

## Abstract

**Objective:**

This study was conducted to determine catalytic properties of wheat phytase with exopolyphosphatase activity toward medium-chain and long-chain inorganic polyphosphate (polyP) substrates for comparative purpose.

**Methods:**

Exopolyphosphatase assay of wheat phytase toward polyP75 (medium-chain polyP with average 75 phosphate residues) and polyP1150 (long-chain polyP with average 1150 phosphate residues) was performed at pH 5.2 and pH 7.5. Its activity toward these substrates was investigated in the presence of Mg^2+^, Ni^2+^, Co^2+^, Mn^2+^, or ethylenediaminetetraacetic acid (EDTA). Michaelis constant (K_m_) and maximum reaction velocity (V_max_) were determined from Lineweaver-Burk plot with polyP75 or polyP1150. Monophosphate esterase activity toward *p*-nitrophenyl phosphate (*p*NPP) was assayed in the presence of polyP75 or polyP1150.

**Results:**

Wheat phytase dephosphorylated polyP75 and polyP1150 at pH 7.5 more effectively than that at pH 5.2. Its exopolyphosphatase activity toward polyP75 at pH 5.2 was 1.4-fold higher than that toward polyP1150 whereas its activity toward polyP75 at pH 7.5 was 1.4-fold lower than that toward polyP1150. Regarding enzyme kinetics, K_m_ for polyP75 was 1.4-fold lower than that for polyP1150 while V_max_ for polyP1150 was 2-fold higher than that for polyP75. The presence of Mg^2+^, Ni^2+^, Co^2+^, Mn^2+^, or EDTA (1 or 5 mM) exhibited no inhibitory effect on its activity toward polyP75. Its activity toward polyP1150 was inhibited by 1 mM of Ni^2+^ or Co^2+^ and 5 mM of Ni^2+^, Co^2+^, or Mg^2+^. Ni^2+^ inhibited its activity toward polyP1150 the most strongly among tested additives. Both polyP75 and polyP1150 inhibited the monophosphate esterase activity of wheat phytase toward *p*NPP in a dose-dependent manner.

**Conclusion:**

Wheat phytase with an unexpected exopolyphosphatase activity has potential as a therapeutic tool and a next-generational feed additive for controlling long-chain polyP-induced inappropriate inflammation from *Campylobacter jejuni* and *Salmonella typhimurium* infection in public health and animal husbandry.

## INTRODUCTION

Inorganic polyphosphate (polyP) is a highly negative-charged linear polymer with three to over a thousand ortho-phosphate (Pi) residues connected by phosphoanhydride bonds like high-energy phosphate compound adenosine 5′-triphosphate [1,2]. It has been reported to be detectable in nearly all biological systems [2]. As a representative example, medium-chain polyP with about 60 to 100 Pi units long has been found in human platelets while long-chain polyP with thousands of Pi units long exists exclusively in infectious microorganisms [2]. In eukaryotes, polyP covers various physiological roles such as bone mineralization, cell proliferation, tumor metastasis, blood clotting and inflammation [1]. However, important roles of polyP in bacteria are concentrated on the expression of pathogenesis [3,4]. In this regard, *Campylobacter jejuni* and *Salmonella typhimurium* are main bacterial pathogens of foodborne gastroenteritis in human all over the world [3,4]. They are closely associated with poultry and its products such as egg, meat, and processed carcass [5–7]. Furthermore, *Campylobacter jejuni* and *Salmonella typhimurium* are ubiquitous in poultry, cattle, swine and sheep, resulting in substantial foodborne illnesses [8]. Indeed, various biological tools that directly or indirectly inactivate these bacteria within the host have been used to decrease the outbreaks of foodborne pathogens in poultry and livestock [8]. Interestingly, *Campylobacter jejuni* and *Salmonella typhimurium* operate well-established polyP metabolic pathways in which polyP pools are principally regulated by exopolyphosphatase hydrolyzing polyP to inorganic phosphate [3,4,9]. Moreover, degradation of polyP can lead to decreased production of their virulent elements and infectious performances in host [3,4].

Today, microbial phytase is one of representative feed enzymes widely employed in animal husbandry [10]. It exclusively catalyzes the release of inorganic phosphate from phytate (inositol-hexakisphosphate, InsP_6_) as its main substrate that is rich in cereals, oilseeds, and legumes [10]. Phytate acts as anti-nutritional agent in mono-gastric animals such as swine and poultry, further generating environmental phosphorus pollution [10]. Intriguingly, wheat phytase is an unusual phytate-degrading enzyme categorized into multiple inositol polyphosphate phosphatase that can non-specifically dephosphorylate *p*-nitrophenyl phosphate (*p*NPP) and 2,3-bisphosphoglycerate as well as more phosphorylated diphospho-myo-inositol pentakisphosphate (PP-InsP_5_) and bisdiphospho-myo-inositol tetrakisphosphate ([PP]_2_-InsP_4_) [11,12]. Thus, wheat phytase might be able to catalyze the hydrolysis of polyP. The objective of this study was to determine catalytic properties of wheat phytase with exopolyphosphatase activity toward medium-chain and long-chain polyP substrates for comparative purpose. To the best of our knowledge, this is the first report about such properties of wheat phytase.

## MATERIALS AND METHODS

### Substrates and preparation of enzyme

Medium-chain polyP (polyP75) with average 75 Pi units long and long-chain polyP (polyP1150) with average 1150 Pi units long were purchased from Kerafast (Boston, MA, USA). The *p*NPP was obtained from Sigma-Aldrich (St. Louis, MO, USA).

Wheat phytase (Sigma-Aldrich, USA) was reconstituted in endotoxin-free water (Sigma-Aldrich, USA). The enzyme was then dialyzed against 50 mM Tris-HCl (pH 8.0) at 4°C overnight. Its inorganic phosphate background was removed by using Pi-bond resin (Innova Biosciences, Cambridge, UK) according to the manufacturer’s instructions.

### Dephosphorylation of inorganic polyphosphates at specific pH

Exopolyphosphatase assay was performed at 37°C for 20 min in 1 mL reaction mixture consisting of 20 μL enzyme, 8 μM polyP75 or polyP1150, and 50 mM Na-acetate (pH 5.2) or Tris-HCl (pH 7.5). Inorganic phosphate release was measured at optical density (OD) 635 nm using a malachite green-based PiColor Lock gold phosphate detection kit (Innova Biosciences, USA) according to the manufacturer’s instructions.

### Effect of additives on exopolyphosphatase activity

Exopolyphosphatase activity was determined at 37°C for 20 min in 1 mL reaction mixture consisting of 20 μL enzyme, 8 μM polyP75 or polyP1150, and 50 mM Tris-HCl (pH 7.5) in the presence of 1 or 5 mM of each additive (Mg^2+^, Ni^2+^, Co^2+^, Mn^2+^, and ethylenediaminetetraacetic acid [EDTA]). Inorganic phosphate release was measured at OD 635 nm using the malachite green-based PiColor Lock gold phosphate detection kit (Innova Biosciences, USA) according to the manufacturer’s instructions.

### Evaluation of enzyme kinetics for exopolyphosphatase activity

Kinetic parameters such as Michaelis constant (K_m_) and maximum reaction velocity (V_max_) for exopolyphosphatase activity were determined from Lineweaver-Burk plot with different concentrations of polyP75 or polyP1150 (8 to 26 μM) at pH 7.5 and 37°C.

### Effect of inorganic polyphosphates on monophosphate esterase activity

Monophosphate esterase activity was assayed at 37°C for 10 min in 0.6 mL reaction mixture containing 20 μL enzyme, 0.25 mM *p*NPP, and 50 mM Na-acetate (pH 5.2) in the presence of polyP75 or polyP1150 (0.01 to 2 mM). The reaction was quenched by adding 0.6 mL of 0.5 M NaOH. Then *p*-nitrophenol release was measured at OD 405 nm.

### Statistical analysis

Statistical analysis was performed by one-way analysis of variance using PROC general linear model (SAS 9.4, SAS Institute Inc, Cary, NC, USA) to test for significant differences between treatments with the Duncan’s multiple range test. The probability levels used for statistical significance were p<0.05. The results were presented as the mean and standard error from three experiments (n = 3).

## RESULTS

### Exopolyphosphatase activity of wheat phytase at specific pH

**Figure 1:**
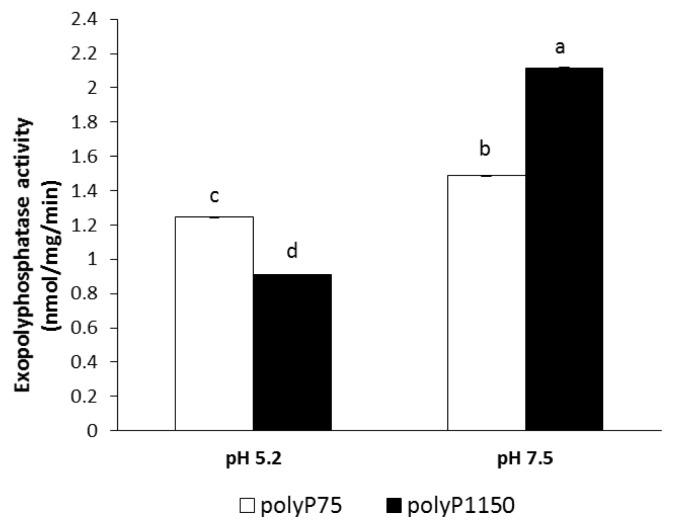
Exopolyphosphatase activity of wheat phytase toward polyP75 and polyP1150 at specific pH. Data were expressed as the mean and standard error from three experiments. PolyP, polyphosphate. ^a–d^ Means with different superscripts differ (p<0.05).

As shown in Figure 1, wheat phytase could dephosphorylate polyP75 and polyP1150 at pH 7.5 more effectively than that at pH 5.2. In addition, its exopolyphosphatase activity toward polyP75 at pH 5.2 was 1.4-fold higher than that toward polyP1150 whereas its activity toward polyP75 at pH 7.5 was 1.4-fold lower than that toward polyP1150.

### Determination of kinetic parameters for exopolyphosphatase activity

Data on enzyme kinetics for exopolyphosphatase activity of wheat phytase were presented in Table 1. The value of K_m_ for polyP75 was 1.4-fold lower than that for polyP1150. Thus, the enzyme showed better substrate affinity against polyP75. Meanwhile, the value of V_max_ for polyP1150 was 2-fold higher than that for polyP75.

### Effect of additives on exopolyphosphatase activity

**Figure 2:**
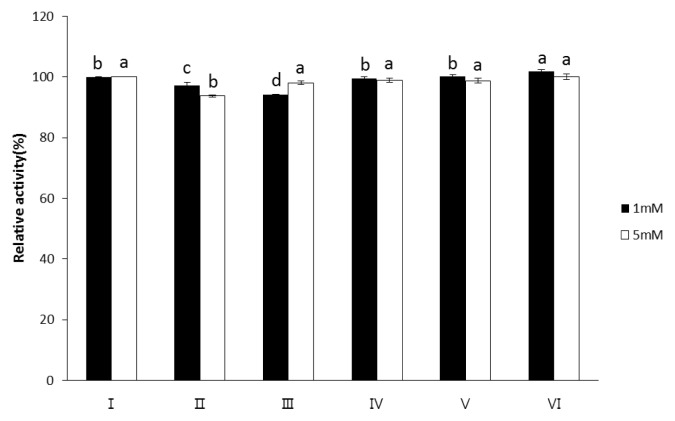
Effect of additives (1 mM or 5 mM) on dephosphorylation of polyP75 by wheat phytase. I: No additive, II: Mn^2+^, III: Ni^2+^, IV: Mg^2+^, V: Co^2+^, VI: ethylenediaminetetraacetic acid. Data were presented as the mean and standard error from three experiments. PolyP, polyphosphate. ^a–d^ Means lacking common superscripts within each concentration differ (p<0.05).

**Figure 3:**
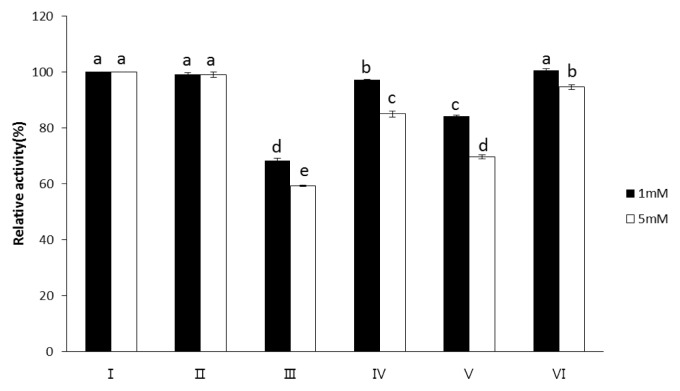
Effect of additives (1 mM or 5 mM) on dephosphorylation of polyP1150 by wheat phytase. I: No additive, II: Mn^2+^, III: Ni^2+^, IV: Mg^2+^, V: Co^2+^, VI: ethylenediaminetetraacetic acid. Data were presented as the mean and standard error from three experiments. PolyP, polyphosphate. ^a–e^ Means lacking common superscripts within each concentration differ (p<0.05).

As shown in Figure 2, the presence of Mg^2+^, Ni^2+^, Co^2+^, Mn^2+^, or EDTA (1 and 5 mM) almost exhibited no inhibitory effect on the exopolyphosphatase activity of wheat phytase toward polyP75. However, the exopolyphosphatase activity toward polyP1150 was inhibited by 1 mM of Ni^2+^ or Co^2+^ (Figure 3). Its activity was also inhibited by 5 mM of Ni^2+^, Co^2+^, or Mg^2+^ (Figure 3). Particularly, Ni^2+^ at 1 mM and 5 mM most strongly inhibited its exopolyphophatase activity toward polyP1150, leading to 32% and 41% loss of its activity, respectively (Figure 3).

### Effect of inorganic polyphosphates on monophosphate esterase activity

**Figure 4:**
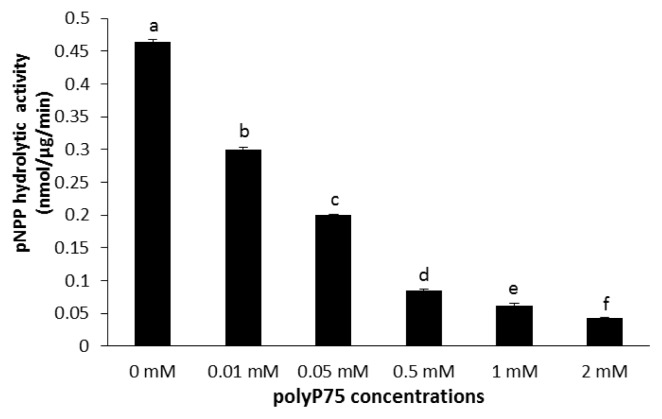
Effect of polyP75 on monophosphate esterase activity of wheat phytase toward p-nitrophenyl phosphate. Data were expressed as the mean and standard error from three experiments. PolyP, polyphosphate. ^a–f^ Means with different superscripts differ (p<0.05).

**Figure 5:**
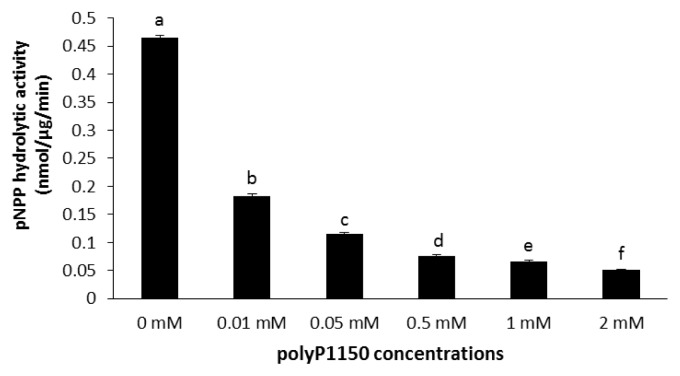
Effect of polyP1150 on monophosphate esterase activity of wheat phytase toward *p*-nitrophenyl phosphate. Data were expressed as the mean and standard error from three experiments. PolyP, polyphosphate. ^a–f^ Means with different superscripts differ (p<0.05).

Both polyP75 and polyP1150 inhibited the monophosphate esterase activity of wheat phytase toward *p*NPP (Figures 4, 5). Furthermore, at the lowest concentration of 0.01 mM, polyP75 and polyP1150 decreased its activity by 35% and 61%, respectively. At 2 mM, they resulted in 90% loss of its activities (Figures 4, 5).

## DISCUSSION

In the present study, catalytic properties of wheat phytase for dephosphorylating long-chain polyP (polyP1150) and medium-chain polyP (polyP75) were described. This study reports novel and significant finding. Up to date, very limited information is available about catalytic properties of polyP degrading enzymes. Previous studies were focused on its hydrolysis of structurally-simple short and medium-chain polyP [9,13].

The exopolyphosphatase activity of wheat phytase toward polyP is unusual in some aspects. Despite the fact that wheat phytase exhibits the highest activity toward phytate and *p*NPP at acidic pH (4.5 to 5.0), it has no activity at pH 7.5 [11], The exopolyphosphatase activity of this enzyme toward polyP75 and polyP1150 was observed at pH 5.2 and 7.5 (Figure 1). On the other hand, calf intestinal alkaline phosphatase previously known as a sole higher eukaryotic-derived enzyme with exopolyphosphatase activity could not degrade polyP15 or polyP75 at pH below 6.0 at all and 90% of its maximal activity was lost at pH 7 to 7.5 [13]. Intriguingly, the catalytic action mode of exopolyphosphatases against polyP substrates at acidic and neutral pH may be different because numbers of Pi residues within substrates required for binding to the enzyme at acidic and neutral pH are 2 and 3, respectively [13]. In particular, wheat phytase was kinetically favorable in degrading long-chain polyP because its overall catalytic efficiency (V_max_/K_m_) for polyP1150 was 1.46-fold higher than that for polyP75 (Table 1). However, values of K_m_ and V_max_ for polyP75 of wheat phytase were 100-fold higher and 250-fold lower, respectively, than those for polyP77 (almost identical to polyP75 in length) of calf intestinal alkaline phosphatase [13]. Thus, the kinetic performance for the exopolyphosphatase activity of wheat phytase toward medium-chain polyP seems to be poor. The exopolyphosphatase activity of wheat phytase toward polyP75 was almost unaffected by the presence of divalent metal ions such as Mg^2+^, Ni^2+^, Co^2+^, or Mn^2+^ (Figure 2). Its activity toward polyP1150 was inhibited by increasing the concentration of Ni^2+^, Co^2+^, and Mg^2+^ (Figure 3). Additionally, EDTA, a well-known metal remover, showed negligible effect on the exopolyphosphatase activity of wheat phytase (Figures 2, 3), suggesting that divalent metal ions could not act as cofactors for its catalytic activity. In previous studies, the exopolyphosphatase activity of inorganic pyrophosphatase from cattle tick, *Rhipicephalus microplus*, relied on Mg^2+^ [9] whereas activities of calf intestinal alkaline phosphatase and soluble pyrophosphatases from protozoa such as *Trypanosoma brucei* and *Leishmania amazonesis* required Zn^2+^ as cofactors [13–15]. The monophosphate esterase activity of wheat phytase toward *p*NPP was inhibited by the presence of polyP75 or polyP1150 in a dose-dependent manner (Figures 4, 5). Thus, it appears that the active site for polyP substrates of wheat phytase is consistent with that for *p*NPP [13]. Similar observation was also made with calf intestinal alkaline phosphatase, displaying competitive inhibitory fashion of *p*NPP-degrading activity by polyP [13].

So far, chickens have been regarded as the primary reservoir of *Campylobacter jejuni* and *Salmonella typhimurium* infection [7,16]. Moreover, long-chain polyP molecules like polyP1150 secreted by these enteric bacteria can aggravate inflammation in hosts such as human and bird [17], leading to intestinal mucosal damage, inflammatory diarrhea, and enteric fever [16,18]. Such situation can severely decrease the productivity of poultry industry and pose public health concern [7]. In conclusion, wheat phytase with an unexpected exopolyphosphatase activity has potential as a therapeutic tool and a next-generational feed additive for controlling long-chain polyP-induced inappropriate inflammation from *Campylobacter jejuni* and *Salmonella typhimurium* infection in public health and animal husbandry because wheat phytase is a relatively safe and endogenous strategy that can overcome drawbacks caused by resistance to antibiotics [6,19] and prejudice and mistrust against the use of foreign microbial enzymes [20].

## Figures and Tables

**Table 1:** Kinetic parameters for exopolyphosphatase activity of wheat phytase

Substrates	K_m_ (μM)	V_max_ (nmol/mg/min)
polyP75	50.86±3.86^b^	9.99±0.54^b^
polyP1150	69.77±1.05^a^	20.08±0.21^a^

Values were expressed as the mean and standard error from three experiments.

K_m_, Michaelis constant; V_max_, maximum reaction velocity; PolyP, polyphosphate.

a,b Means with different superscripts within column differ (p<0.05).
